# A Large Benign Adrenocortical Adenoma Cosecreting Testosterone and Cortisol

**DOI:** 10.1210/jcemcr/luae045

**Published:** 2024-04-24

**Authors:** Martha Dillon, Sara Shteyman, Samaneh Rabiehashemi, Parvathy Madhavan, Pooja Luthra

**Affiliations:** Division of Endocrinology and Metabolism, University of Connecticut School of Medicine, Farmington, CT 06030, USA; Division of Endocrinology and Metabolism, University of Connecticut School of Medicine, Farmington, CT 06030, USA; Division of Endocrinology and Metabolism, University of Connecticut School of Medicine, Farmington, CT 06030, USA; Division of Endocrinology and Metabolism, Hartford Hospital, Hartford, CT 06106, USA; Division of Endocrinology and Metabolism, University of Connecticut School of Medicine, Farmington, CT 06030, USA; Division of Endocrinology and Metabolism, University of Connecticut School of Medicine, Farmington, CT 06030, USA

**Keywords:** adrenal adenoma, Cushing syndrome, hypercortisolism, hyperandrogenism, virilization, adrenocortical carcinoma

## Abstract

Most adrenal incidentalomas are benign neoplasms of the adrenal cortex. While the majority are nonfunctional, many secrete cortisol. Androgen- or estrogen-secreting adenomas are rare. A 44-year-old female, with history of hypertension and prediabetes, presented with worsening acne, hirsutism, secondary amenorrhea for 2 years, and a 40-pound weight gain. Laboratory evaluation showed high 24-hour urine free cortisol, suppressed adrenocorticotropic hormone (ACTH) level, indicative of ACTH independent Cushing syndrome, and elevated testosterone and androstenedione. Abdominal computed tomography (CT) revealed a 6.3 × 5.2 × 5.6 cm left adrenal mass. Patient underwent left open adrenalectomy. Pathology revealed benign adrenocortical adenoma. Postoperatively there was a significant improvement in her blood pressure and blood sugar levels, resumption of menses, and complete resolution of hyperandrogenism and hypercortisolism. We describe a patient with an adrenal adenoma cosecreting cortisol and androgen, leading to Cushing syndrome and significant virilization. Adrenal masses secreting androgens are less common and concerning for adrenocortical carcinoma (ACC). Patients with adrenal masses cosecreting multiple hormones should undergo workup expediently since ACC confers poor outcomes.

## Introduction

Adrenal incidentalomas have a prevalence of 3% in the middle-aged population and as high as 10% by the age of 70 years (1, 2). Most incidentalomas are benign, nonfunctioning neoplasms of the adrenal cortex. Functioning adrenal adenomas frequently secrete cortisol or aldosterone. Secretion of androgens or estrogens or cosecretion of both cortisol and androgens is very rare (2). We present a patient with a large benign adrenal adenoma cosecreting cortisol and testosterone.

## Case Presentation

The patient is a 44-year-old woman with history significant for hypothyroidism secondary to radioactive iodine treatment for Graves disease, hypertension, and prediabetes. She had a 2-year history of worsening acne, fullness of her face, male pattern hair loss, coarse abdominal hair, purple abdominal striae, secondary amenorrhea, and 40-pound weight gain. Her periods had previously been irregular. She had a strong family history of non-Hodgkin lymphoma involving several first- and third-degree maternal relatives, and she had several second- and third-degree paternal relatives with leukemia, intra-abdominal cancers, and intracranial malignancies. On examination, blood pressure was 150/97 mmHg and body mass index was 33.99 kg/m^2^. She had significant hirsutism, supra-clavicular fullness, facial plethora, dorsocervical fullness, central obesity, thick purplish abdominal striae, and proximal muscle weakness.

## Diagnostic Assessment

**Figure 1.**
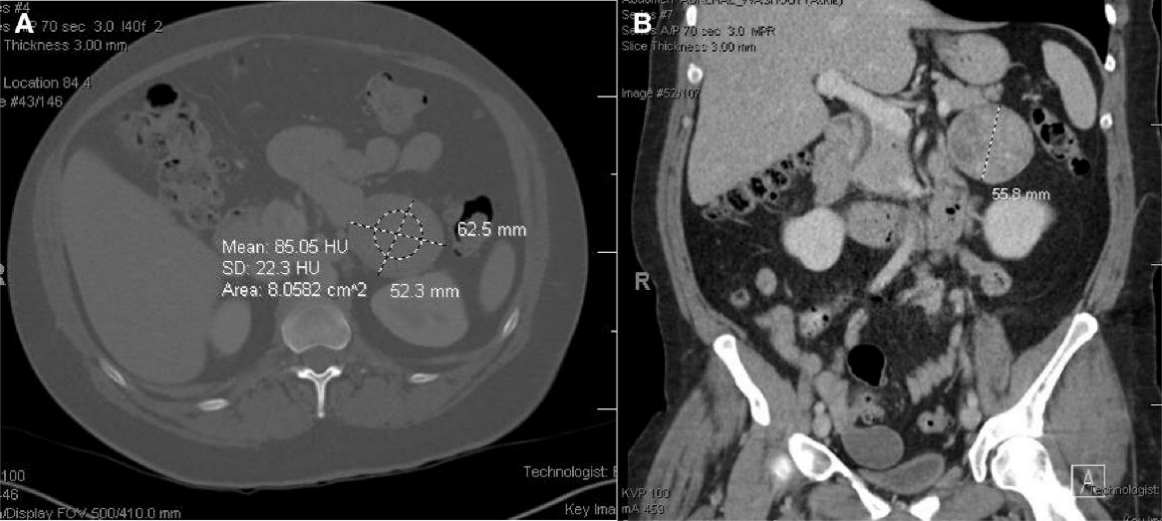
Axial (A) and coronal (B) computed tomography of the abdomen with IV contrast demonstrating left adrenal mass. The study revealed a 6.3 × 5.2 × 5.6 cm left adrenal mass. It measured 22 Hounsfield units (HU) on noncontrast imaging, 85 HU on 70-second imaging, and 48 HU on 15-minute delayed phase. Calculated relative washout was 43.5% and absolute washout was 59.4%. Additionally, there was another 1.1-cm low-density nodule in the on left adrenal genu. The right adrenal gland also had a 1.2-cm low-density nodule with 0 HU on all phases compatible with a lipid-rich adenoma.

Baseline laboratory studies showed normal basic metabolic profile, ACTH level < 5 pg/mL (< 1.1 pmol/L) (normal reference range, 7.2–63.3 pg/mL; 1.58–13.92 pmol/L), serum cortisol at 2 Pm 18.1 mcg/dL (499.56 nmol/L) (normal reference range, 7–23 mcg/dL; 193.2–634.8 nmol/L), total testosterone 152 ng/dL (5.27 nmol/L) (normal reference range, 9–55 ng/dL; 0.31–1.91 nmol/L) and androstenedione 2.72 ng/mL (9.49 nmol/L) (normal reference range, 0.13–0.82 ng/mL; 0.45–2.86 nmol/L). Dehydroepiandrosterone (DHEA) 0.74 ng/mL (2.57 nmol/L) (normal reference range, 0.63–4.70 ng/mL; 2.18–16.29 nmol/L) and dehydroepiandrosterone sulfate (DHEAS) 65.00 mcg/dL (1.76 mcmol/L) (normal reference range, 32.00–240.00 mcg/dL; 0.86–6.48 mcmol/L) were normal. Plasma free metanephrines and normetanephrines were also normal. The 24-hour urinary free cortisol was elevated at 169.32 mcg/day (467.32 nmol/day) (normal reference range, ≤ 45.00 mcg/day; ≤ 124.20 nmol/day), which standardized to 107.1 mcg/g of creatinine (33.38 nmol/mmol creatinine) (normal reference range, less than 24 mcg/g creatinine; less than 7.49 nmol/mmol creatinine) (Table 1). CT of the abdomen and pelvis with and without contrast showed a 6.3 × 5.3 × 5.6 cm left adrenal mass, 22 Hounsfield units (HU) on noncontrast imaging, 85 HU on 70 second imaging, and 48 HU on 15-minute delayed phase. Calculated relative washout was 43.5% and absolute washout was 59.4%. There was another 1.1-cm low-density nodule in the left adrenal genu and a 1.2-cm low-density nodule in the right adrenal gland consistent with a lipid-rich adenoma (Fig. 1). Pelvic ultrasound revealed a 2.5-cm simple right ovarian cyst and a 1.3-cm intramural uterine fibroid. None of these were thought to be the source of the significant hyperandrogenism.

**Table 1.** Hormonal concentrations in our patient on presentation

Hormone tested	Value	Reference range
Plasma metanephrine	< 19.01 pg/mL (< 100.00 pmol/L)	0.00–93.16 pg/mL (0.00–490 pmol/L)
Plasma normetanephrine	65.93 pg/mL (360 pmol/L)	0.00–163.00 pg/mL (0.00–890 pmol/L)
Prolactin	16.56 ng/mL (16.56 mcg/L)	2.74–19.64 ng/mL (2.74–19.64 mcg/L)
Thyroid stimulating hormone	0.33 uIU/mL (0.33 IU/L)	0.35–4.94 uIU/mL (0.35–4.94 IU/L)
Aldosterone	4.30 ng/dL (0.12 nmol/L)	< 31.00 ng/dL (< 0.86 nmol/L)
Renin activity	1.3 ng/mL/h (1.3 mcg/L/h)	0.2–1.6 ng/mL/h (0.2–1.6 mcg/L/h)
Adrenocorticotropic hormone	**<** **5.00 pg/mL (< 1.10 pmol/L)**	7.20–63.30 pg/mL (1.58–13.92 pmol/L)
Cortisol (2:00 Pm)	**18.10 mcg/dL (499.56 nmol/L)**	7.00–23.00 mcg/dL (193.20–634.80 nmol/L)
Cortisol, urine free	**169.32 mcg/day (467.32 nmol/day)**	≤ 45.00 mcg/day (≤ 124.20 nmol/day)
Creatinine, 24-hour urine	1.48 g/day (13.08 mmol/day)	0.78–2.31 g/day (6.90–20.42 mmol/day)
Cortisol, urine free/creatinine	**107.10 mcg/g creatinine (33.38 nmol/mmol creatinine)**	< 24.00 mcg/g creatinine (< 7.49 nmol/mmol creatinine)
Follicle stimulating hormone	3.44 mIU/mL (3.44 IU/L)	16.74–113.59 mIU/mL (16.74–113.59 IU/L)
Luteinizing hormone	1.51 mIU/mL (1.51 IU/L)	10.87–58.64 mIU/mL (10.87–58.64 IU/L)
DHEA	0.74 ng/mL (2.57 nmol/L)	0.63–4.70 ng/mL (2.18–16.29 nmol/L)
DHEA sulfate	65.00 mcg/dL (1.76 mcmol/L)	32.00–240.00 mcg/dL (0.86–6.48 mcmol/L)
Estradiol	49.00 pg/mL (179.89 pmol/L)	<20.00–40.00 pg/mL (< 73.40–146.80 pmol/L)
Estrone	64.30 pg/mL (237.82 pmol/L)	3.00–32.00 pg/mL (11.09–118.34 pmol/L)
Sex hormone binding globulin	**1.24 mcg/mL (13.00 nmol/L)**	2.85–12.83 mcg/mL (30.00–135.00 nmol/L)
Testosterone, LC-MS/MS	**152.00 ng/dL**	
	**(5.27 nmol/L)**	9.00–55.00 ng/dL (0.31–1.91 nmol/L)
Testosterone, free	**38.60 pg/mL (133.83 pmol/L)**	1.10–5.80 pg/mL (3.81–20.11 pmol/L)
Androstenedione	**2.72 ng/mL (9.49 nmol/L)**	0.13–0.82 ng/mL (0.45–2.86 nmol/L)
Hemoglobin A1c	6.0%	4.4%–6.4%

Abnormal values shown in bold font. Values in parenthesis are International System of Units (SI). Abbreviations: DHEA, dehydroepiandrosterone. LC-MS/MS, liquid chromatography/tandem mass spectrometry.

## Treatment

**Figure 2.**
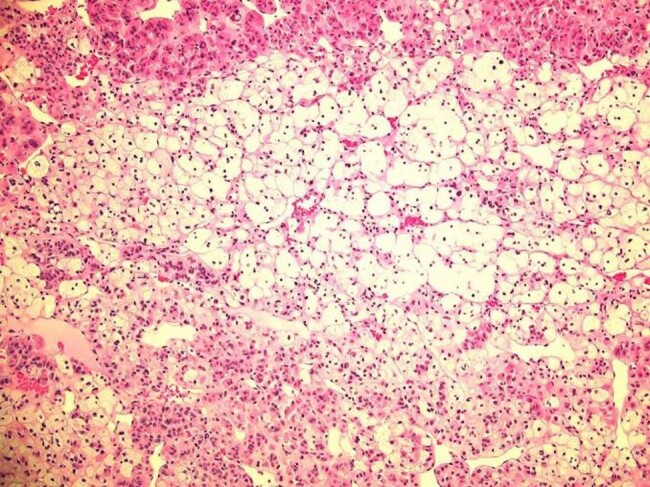
Adrenal cortical adenoma. Hematoxylin and eosin stain. Large expanse of enlarged cells with lipid-rich cytoplasm (clear cells). Heterogeneity of tumor cell types, clear and amphophilic.

Patient was referred for genetic testing; however, it was determined she did not fit the pattern for Li-Fraumeni syndrome or pathogenic *RUNXq* mutations and did not meet National Comprehensive Cancer Network (NCCN) criteria for genetic testing (3). She underwent left open adrenalectomy, which showed no gross invasion into adjacent tissues. Pathology revealed a 6-cm adrenocortical adenoma with no necrosis, extra-adrenal invasion, or vascular invasion. Immunohistochemical staining showed a large expanse of enlarged cells with lipid-rich cytoplasm (clear cells) (Fig. 2). The MIB-1 index was less than 1%. She was discharged on prednisone 10 mg twice daily with a gradual taper and discontinuation of steroids over the next 4 months.

## Outcome and Follow-Up

**Figure 3.**
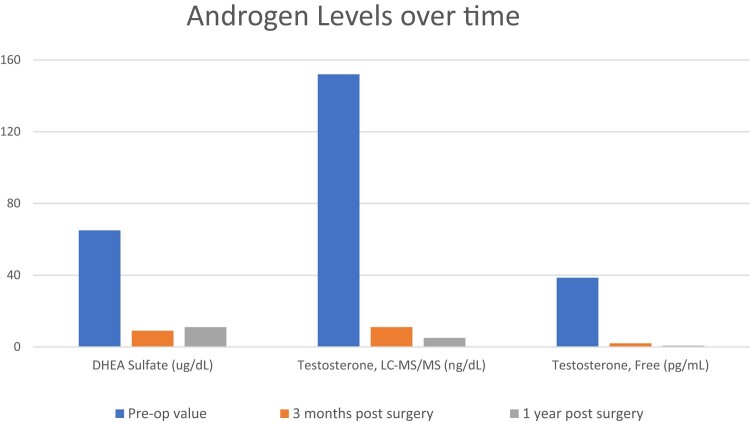
Androgen levels before and after surgery at 3 months and 1 year. All androgen markers were noted to normalize after adrenalectomy.

Postoperatively, the patient had resolution of hypertension, prediabetes, resumption of regular menses within 2 months, improvement in hair loss, and a weight loss of 60 pounds. Hormonal testing at 3 months and 1 year after surgery demonstrated resolution of hyperandrogenism and hypercortisolism (Fig. 3, Table 2). Repeat CT abdomen 1 year after surgery showed a stable 1.2-cm nodule in the right adrenal gland of 9 HU, consistent with benign adenoma. Four years after surgery, hormone levels continue to be within reference range (Table 2).

**Table 2.** Postoperative hormone concentrations at multiple time points in our patient

Hormone tested	3 months postsurgery	1 year postsurgery	4 years postsurgery	Reference range
Plasma normetanephrine	N/A	N/A	40.00 pg/mL (218.40 pmol/L)	0.00–218.90 pg/mL (0.00–1195.19 pmol/L)
Plasma metanephrine	N/A	N/A	< 10.00 pg/mL (< 52.00 pmol/L)	0.00–88.00 pg/mL (0.00–462.88 pmol/L)
Thyroid stimulating hormone	1.04 uIU/mL (1.04 IU/L)	2.08 uIU/mL (2.08 IU/L)	5.07 uIU/mL (5.07 IU/L)	0.35–4.94 uIU/mL (0.35–4.94 IU/L)
Adrenocorticotropic hormone	N/A	12.20 pg/mL (2.68 pmol/L)	13.00 pg/mL (2.86 pmol/L)	7.20–63.30 pg/mL (1.58–13.92 pmol/L)
Cortisol (AM)	N/A	6.70 mcg/dL (184.92 nmol/L)	N/A	7.00–23.00 mcg/dL (193.20–634.80 nmol/L)
Cortisol (AM) after 1-mg dexamethasone suppression test	N/A	N/A	**1.60 mcg/dL (44.16 nmol/L)**	6.20–19.40 mcg/dL (171.12–535.44 nmol/L)
Dexamethasone	N/A	N/A	226.00 ng/dL (5.76 nmol/L)	140.00–295.00 ng/dL (3.57–7.52 nmol/L)
Salivary cortisol, midnight day 1	N/A	N/A	**0.14 mcg/dL (3.81 nmol/L)**	< 0.01–0.09 mcg/dL (< 0.28–2.48 nmol/L)
Salivary cortisol, midnight day 2	N/A	N/A	**0.22 mcg/dL (6.15 nmol/L)**	< 0.01–0.09 mcg/dL (< 0.28–2.48 nmol/L)
Salivary cortisol, midnight day 3	N/A	N/A	0.08 mcg/dL (2.29 nmol/L)	< 0.01–0.09 mcg/dL (< 0.28–2.48 nmol/L)
Cortisol, urine free	< 2.80 mcg/day (< 7.73 nmol/day)	N/A	19.00 mcg/day (52.44 nmol/day)	≤ 45.00 mcg/day (≤ 124.20 nmol/day)
Creatinine, 24-hour urine	1.26 g/day (11.14 mmol/day)	N/A	1.40 g/day (12.38 mmol/day)	0.78–2.31 g/day (6.90–20.42 mmol/day)
Cortisol, urine free/creatinine	< 2.13 mcg/g creatinine (<0.66 nmol/mmol creatinine)	N/A	13.60 mcg/g creatinine (4.24 nmol/mmol creatinine)	< 24.00 mcg/g creatinine (< 7.49 nmol/mmol creatinine)
Follicle stimulating hormone	28.83 mIU/mL (28.83 IU/L)	3.72 mIU/mL (3.72 IU/L)	N/A	16.74–113.59 mIU/mL (16.74–113.59 IU/L)
Luteinizing hormone	25.15 mIU/mL (25.15 IU/L)	7.40 mIU/mL (7.40 IU/L)	N/A	10.87–58.64 mIU/mL (10.87–58.64 IU/L)
DHEA sulfate	**9.00 mcg/dL (0.24 mcmol/L)**	**11.00 mcg/dL (0.30 mcmol/L)**	N/A	32.00–240.00 mcg/dL (0.86–6.48 mcmol/L)
Estradiol	< 20.00 pg/mL (< 73.40 pmol/L)	66.00 pg/mL (242.22 pmol/L)	N/A	< 20.00–40.00 pg/mL (< 73.40–146.80 pmol/L)
Sex hormone binding globulin	2.66 mcg/mL (28.00 nmol/L)	3.80 mcg/mL (40.00 nmol/L)	N/A	2.85–12.83 mcg/mL (30.00–135.00 nmol/L)
Testosterone, LC-MS/MS	11.00 ng/dL (0.38 nmol/L)	5.00 ng/dL (0.17 nmol/L)	4.20 ng/dL (0.15 nmol/L)	9.00–55.00 ng/dL (0.31–1.91 nmol/L)
Testosterone, free	2.00 pg/mL (6.93 pmol/L)	0.70 pg/mL (2.43 pmol/L)	N/A	1.10–5.80 pg/mL (3.81–20.11 pmol/L)
Androstenedione	0.68 ng/mL (2.39 nmol/L)	N/A	0.35 ng/mL (1.22 nmol/L)	0.13–0.82 ng/mL (0.45–2.86 nmol/L)
Hemoglobin A1c	5.1%	5.2%	5.6%	4.4%–6.4%

Abnormal values shown in bold font. Values in parenthesis are International System of Units (SI). Abbreviations: DHEA, dehydroepiandrosterone; LC-MS/MS; liquid chromatography/tandem mass spectrometry; N/A, not available.

## Discussion

We present a patient with a benign adrenocortical adenoma cosecreting cortisol and testosterone. In contrast to our patient, most adrenal adenomas are nonfunctioning. Functional adrenal adenomas usually secrete cortisol or aldosterone (4). The incidence of Cushing syndrome varies from 0.7 to 2.4 per million population per year. Based on recent literature, 15.6% of cases of Cushing syndrome are due to adrenal adenomas and 6% due to ACC (5). Conversely, virilizing tumors are more likely to be malignant (4). ACC is rare, with an annual incidence estimated between 0.5 and 2 cases per million population (6). The average age at diagnosis ranges from 47 to 55 years (7, 8). Women with excess androgen secretion present with hirsutism, clitoromegaly, and menstrual irregularities. Androgen excess is often under-recognized in male patients due to the lack of clinically apparent symptoms, and they are more likely to present with gynecomastia and hypogonadism due to peripheral conversion of excess androgens into estrogen, leading to suppression of hypothalamic-pituitary-gonadal axis by feedback inhibition and subsequent gonadal atrophy. Interestingly, our patient had elevated androstenedione and testosterone but normal DHEA and DHEAS. Androgen-secreting adrenal tumors often produce multiple androgens, although there are documented cases of adrenal tumors producing only one androgen. Pingle et al described a case of a purely DHEAS-secreting adrenal neoplasm (9). Moreno et al had a case series of 21 androgen-secreting adrenal tumors in women; of the 14 with hormonal testing, all had elevated testosterone, only 1 did not have elevated androstenedione, while 4 did not have elevated DHEAS and 3 did not have elevated DHEA (10). ACTH-independent Cushing syndrome is often accompanied by a reduction in adrenal androgens since hypercortisolism will cause suppression of ACTH secretion from the pituitary due to feedback inhibition, causing atrophy of peritumoral and contralateral adrenal cortex (11). This dissociation of cortisol and androgen production for cortisol-secreting adenomas was thought to be a contrasting trait to the concurrent production of these hormones seen in roughly half of all ACC patients with hormone excess (11, 12). However, Kamenicky et al showed that even benign cortisol-secreting adenomas are able to secrete normal to low normal levels of androgens which typically do not cause virilization (11). There are rare case reports of adenomas that cause both Cushing disease and virilization (13-18).

Numerous molecular mechanisms lead to development of adrenocortical tumors, including genetic alteration in familial tumor syndromes such as the *IGF-II* overexpression in Beckwith-Wiedmann syndrome, *TP53* gene mutations in Li- Fraumeni syndrome, *Menin (11q13)* gene in multiple endocrine neoplasia (MEN) 1, *PRKARIA (17q22-24)* of Carney complex, as well as Lynch syndrome, among others. Sporadic tumors can also have mutations in *Menin, TP53,* and *CYP21* genes (4, 12).

It is extremely important to differentiate a benign adenoma from carcinoma. Imaging techniques such as CT of the abdomen with and without contrast is usually the first imaging modality used for adrenal tumors as tumor size, HU, and absolute and relative washout percentages are important features for determining malignancy. The HU on a noncontrast CT can assess the lipid content of adrenal masses. Malignant lesions do not contain lipids, while many adenomas are lipid rich. Previous guidelines recommended using a cutoff of less than or equal to 10 HU to differentiate adenoma from carcinoma. However, this led to incorrect diagnoses of lipid-poor or complicated (hemorrhagic) adenomas (19). Iniguez-Ariza et al confirmed that attenuation of more than 10 HU is 100% sensitive for malignant adrenal tumors; however, the specificity was only 46%. Additionally, only half of benign adrenal tumors had an attenuation of less than or equal to 10 HU. They recommended using an attenuation threshold of at least 20 HU for a similar sensitivity (98%), with a higher specificity (64%) for malignancy compared to 46% (20). Additionally, adrenal adenomas have high washout on contrast-enhanced CT. Absolute washout above 60% and a relative washout greater than 40% have been proposed to accurately diagnose benign adenomas. Lipid-poor adenomas have similar relative washout percentages, as lipid-rich adenomas and can be distinguished from malignant masses or metastases using this method (19). Magnetic resonance imaging (MRI) can also be useful in determining ACC, which have increased heterogeneity, internal hemorrhagic foci, vascular extension, calcifications, and necrosis (12).

In addition to imaging, there are multiple histopathologic schemas to differentiate a benign adenoma from ACC. The classic classification system of adrenal cortical neoplasms is the modified Weiss histopathologic system (21). This scoring system includes 9 histologic parameters: high nuclear grade of III or IV, a mitotic count greater than 5 per 50 high power fields, atypical mitosis, necrosis, diffuse architecture greater than 33% of tumor volume, clear cells less than or equal to 25% of tumor volume, capsular infiltration, venous invasion, and sinusoidal or lymphatic invasion (22). To be classified as malignant, the neoplasm needs to meet 3 of these criteria (23). The recent 2022 World Health Organization (WHO) guidelines have expanded to include other multiparameter diagnostic algorithms, including the reticulin algorithm, the Lin-Weiss-Bisceglia system, and the Helsinki scoring system (22). The addition of immunohistochemistry has been useful in creating more objective classification. The reticulin algorithm uses silver histochemical staining to assess for an altered reticulin framework in addition to mitotic count, tumor necrosis, and vascular invasion (24). The Ki67 proliferation marker protein is overexpressed in malignant tissue due to the high levels of cellular division and was integrated into ACC diagnosis with the Helsinki score. A Ki67 labeling index measured by immunostaining with its monoclonal antibody MIB-1 (25) of greater than 5% is suspicious for ACC (22, 26). Immunohistochemistry using the MIB-1 index was < 1% in our patient, which was consistent with an adrenal adenoma.

As imaging can be indeterminate or have false positives due to lipid-poor adenomas, current differentiation between adrenal adenoma and ACC can rely on postsurgical histopathology schemes. However, novel noninvasive tests in development may help with diagnosis of ACC. A promising test is the urine steroid metabolomics profile in 24-hour urine samples which use both machine learning data analysis and liquid chromatography–tandem mass spectrometry profiling of 15 different urinary steroid metabolites. The profile includes multiple metabolites, as ACC can overproduce steroid precursors instead of clinically active compounds (27). The recent EURINE-ACT study, published in 2020, included the use of urine metabolomics to aid in the discrimination of ACC from adenomas preoperatively. When combined with tumor diameter and imaging characteristics, including an unenhanced CT tumor attenuation cutoff of 20 HU, the study found a higher positive predictive value for detecting ACC without compromising specificity. They also had lower false positive rates than unenhanced CT, MRI chemical shift, and fludeoxyglucose (^18^F-FDG) positron emission tomography (PET)/CT scan in a prospective study (28).

It is also important to consider other etiologies of hyperandrogenism, such as polycystic ovarian syndrome, congenital adrenal hyperplasia with 21-hydroxylase deficiency, ovarian hyperthecosis, and androgen-producing tumors of the ovary or adrenals (29). Patients with ACC have rapid onset of symptoms and higher levels of DHEAS and testosterone in comparison to other etiologies (11). Our patient did not have any suspicious masses of the ovaries on imaging preoperative imaging and the androgen levels normalized after adrenalectomy (Fig. 2).

In conclusion, we describe a 44-year-old female patient with a benign adrenal adenoma cosecreting cortisol and androgen, causing Cushing syndrome and significant virilization.

## Learning Points

- Adrenal adenomas that secrete androgens or estrogens are more likely to be ACC and rare in benign adenomas.
- CT of the abdomen with and without contrast is usually the first imaging modality used for adrenal tumors as tumor size, HU, and absolute and relative washout percentages are important features for determining the malignant potential of an adenoma.
- When imaging is indeterminate, histopathology after surgical resection can determine if an adenoma is benign or malignant.

## Data Availability

Data sharing is not applicable to this article as no datasets were generated or analyzed during the current study.

## Abbreviations

ACC: adrenocortical carcinoma
ACTH: adrenocorticotropic hormone
CT: computed tomography
DHEA: dehydroepiandrosterone
DHEAS: dehydroepiandrosterone sulfate
HU: Hounsfield units
